# Gait Analysis for Post-Stroke Hemiparetic Patient by Multi-Features Fusion Method

**DOI:** 10.3390/s19071737

**Published:** 2019-04-11

**Authors:** Mengxuan Li, Shanshan Tian, Linlin Sun, Xi Chen

**Affiliations:** State Key Laboratory of Mechatronics Engineering and Control, Beijing Institute of Technology, Beijing 100081, China; formlmx@126.com (M.L.); shanshanbit@126.com (S.T.); sll8686886@163.com (L.S.)

**Keywords:** walking, hemiparetic gait, electrostatic field sensing, gait feature extraction

## Abstract

Walking is a basic requirement for participating in daily activities. Neurological diseases such as stroke can significantly affect one’s gait and thereby restrict one’s activities that are a part of daily living. Previous studies have demonstrated that gait temporal parameters are useful for characterizing post-stroke hemiparetic gait. However, no previous studies have investigated the symmetry, regularity and stability of post-stroke hemiparetic gaits. In this study, the dynamic time warping (DTW) algorithm, sample entropy method and empirical mode decomposition-based stability index were utilized to obtain the three aforementioned types of gait features, respectively. Studies were conducted with 15 healthy control subjects and 15 post-stroke survivors. Experimental results revealed that the proposed features could significantly differentiate hemiparetic patients from healthy control subjects by a Mann–Whitney test (with a p-value of less than 0.05). Finally, four representative classifiers were utilized in order to evaluate the possible capabilities of these features to distinguish patients with hemiparetic gaits from the healthy control subjects. The maximum area under the curve values were shown to be 0.94 by the k-nearest-neighbor (kNN) classifier. These promising results have illustrated that the proposed features have considerable potential to promote the future design of automatic gait analysis systems for clinical practice.

## 1. Introduction

A report from the American Heart Association suggests that 7.2 million Americans have suffered from a stroke, and about 795,000 people experience a new or recurrent stroke each year [1]. In China, the world’s largest developing country, the number of stroke patients is 22.3 million [2]. Furthermore, the literature shows that stroke will continue to be the world’s second leading cause of years of life lost (YLLs) from 2016 to 2040 [3]. Following a stroke, about 10% of patients die, whereas the survivors suffer from partial disability, such as hemiplegia, which can significantly reduce their mobility and damage their independence with regard to performing activities of daily living.

Walking impairment occurs in most stroke survivors. It is characterized by a pronounced clinical presentation of gait asymmetry, as compared to healthy people. A previous study has shown that stroke could reduce the weight-bearing capacity of the lower limbs by up to 43% [4]. Moreover, stroke survivors have a decreased stance phase and a prolonged swing phase on the paretic side [5]. Furthermore, walking speed is decreased and the stride length is shorter [6]. Motor rehabilitation is essential to help patients with hemiplegia recover their motor function. In order to develop appropriate treatment strategies for stroke patients, it is important to accurately acquire, identify and evaluate their gait abnormalities. At present, the most common method of diagnosing and assessing the improvement of patients with hemiplegia still depends on various questionnaires, especially in developing countries. Although traditional assessment questionnaires have been used for many years and the evaluation results have been widely accepted in various fields, the use of questionnaires may lead to subjective results, which are highly dependent on the experience of specialists. Therefore, an objective assessment of patients’ gait abnormalities is of great importance to current clinical practice, and enables specialists to develop a more customized treatment plan and a more scientific assessment of the performance of therapy.

Nowadays, various measurement techniques have made it easier to collect different types of gait data during walking, which greatly facilitates the development of automatic gait analysis. An optical motion capture system (such as VICON) can provide an instantaneous position of markers located on one’s body that can be used to analyze gait [7]. Researchers have also developed the foot plantar pressure system, which consists of sensors mounted under a mat that can be used for gait parameter collection [8]. Although these systems can measure gait parameters accurately, they have the disadvantage of a long setup time, operational complexity due to the specialized technical knowledge, high cost and restriction to lab-based settings. The aforementioned defects greatly restrict the usage of these gait analysis systems.

Thus, researchers have developed wearable sensors to analyze gait that are cost effective and portable. The wearable sensors, such as accelerometers [9], gyroscopes [10], force sensors [11], electromyography [12], etc., can be attached to one’s body for analyzing gait. The inertial measurement units (IMU), which includes combinations of accelerometers and gyroscopes, can be used for gait assessment and is based on the measurement of acceleration and orientation of the foot and limb as used in different studies. Wearable sensors have been used in gait event estimation [13], gait action recognition [14], and gait analysis [15] because they are miniaturized, low powered, durable, inexpensive, and highly mobile. However, these wearable sensors share a common disadvantage; they all require the placement of a device on the subject’s body, which may be obtrusive. Moreover, wireless systems usually store data on storage cards or transmit data through Bluetooth devices to personal computers, which have a high energy demand and cannot undergo signal analysis over a long period of time.

With the rapid development of machine learning techniques, researchers have combined gait data obtained by different types of sensors with feature extraction methods and classification models to implement an automated and accurate diagnosis system for the purpose of clinical assistance. Many previous studies have focused on the recognition problem of gait abnormality, such as [16,17,18,19]. These studies have demonstrated the use of statistical and time-frequency domain methods for feature extraction from gait signals and some classifiers for the automatic diagnosis of an abnormal gait. The features used in the classification can obtain a decent classification performance, but they may not contain exact pathological significance. The features used by the classifier should be related to the characteristics of the disease in order to achieve a better generalization effect.

In our previous study [20], we developed an electrostatic field sensing (EFS) technology, based on the gait analysis method, with the advantage of affordable, wear-free and long-time monitoring, which may be capable of continuously monitoring gait parameters during daily activities. This paper further extends and develops our previous work to a new field: Automatic gait analysis of post-stroke hemiplegia patients. The EFS method can obtain several gait temporal parameters accurately. These parameters, such as gait cycle, stance phase duration, swing phase duration, gait cadence, etc., can intuitively describe gait. However, these temporal parameters cannot explain the fluctuation in the gait signal, which will cause some gait parameters to be neglected. Therefore, several other features, which were seldom applied before, such as gait symmetry, complexity characteristics and stability index, were extracted for analysis. After analysis, these features were fed to four representative classifiers to achieve the final classification results.

The aim of this paper is to propose an automatic method for diagnosing post-stroke disease from the gait information recorded by the EFS method. The rest of this manuscript has been organized into the following sections. In Section 2, we briefly introduce the principle and system of the EFS method, the feature extraction method, the classification model, and the data collection procedures. In Section 3, we show the feature analysis result and the classification accuracy. In Section 4, we discuss the relationship between the proposed characteristics and post-stroke gaits. We conclude the paper in Section 5, with final comments and conclusions.

## 2. Materials and Methods

### 2.1. Subjects

The study recruited 15 post-stroke hemiparetic patients (HP) (nine males, six females) from the Zhongshan People’s Hospital, Guangdong, China, as the pathological group. Their average mass was 72.4 kg (range: 56–78 kg), average height was 1.65 m (range: 1.54–1.74 m), average body mass index was 26.5 (range: 23.6–28.8) and average age was 46 years (range: 31–60 years). At the same time, 15 matched healthy volunteers (HC) (nine males, six females) were enrolled as the normal reference group. Their average mass was 65.7 kg (range: 45–79 kg), average age was 29 years (range: 24–33 years), average body mass index was 22.1 (range: 18.5.6–24.3) and average height was 1.72 m (range: 1.56–1.81 m).

For the pathological group, the inclusion criteria included: (1) First stroke with unilateral hemiparesis as confirmed by computed tomography or magnetic resonance imaging, (2) ability to step or walk for at least 1 min without using any assisted devices, (3) ability to understand external commands and to cooperate with the experimental procedures, (4) no other diseases known to affect gait. For the normal reference group, the exclusion criteria included neurological or lower extremity conditions, respiratory or cardiovascular problems, insanity or mental disorder, and pregnancy. This research was approved by the Ethics Committee of Zhongshan People’s Hospital, and an informed consent form was signed by each subject.

### 2.2. Electrostatic Field Sensing System

The human body is charged due to friction between skin and clothing [21,22,23]. Previous studies have shown that the electric field change of the human body is mainly due to the movement of the foot. Thus, foot movement can be obtained from the change of the electric field around the human body. The detailed principle of the electrostatic field sensing (EFS) method and system installation was described in our previous study [20]. We used the same EFS measurement installation in this study and a brief introduction is as follows: The induction electrode was utilized to sense changes in the electric field generated by the movement of the human foot. The induced current generated by foot movement was then converted to an observable voltage signal from the I–V converter and the operational amplifier. The electric field perturbation caused by human foot movement is a low-frequency electric field with a typical frequency of 1–2 Hz. We designed a low-pass filter with a cutoff frequency of 20 Hz to eliminate the interference of high-frequency electric fields and power frequency of the grid (50 Hz). Thereafter, the analog signal was converted into the digital signal through analog-to-digital conversion (AD) for data acquisition. The sampling rate adopted in this paper was 1 kHz. The digital signal obtained by AD sampling was sent to a personal computer for data processing through a USB cable.

**Figure 1:**
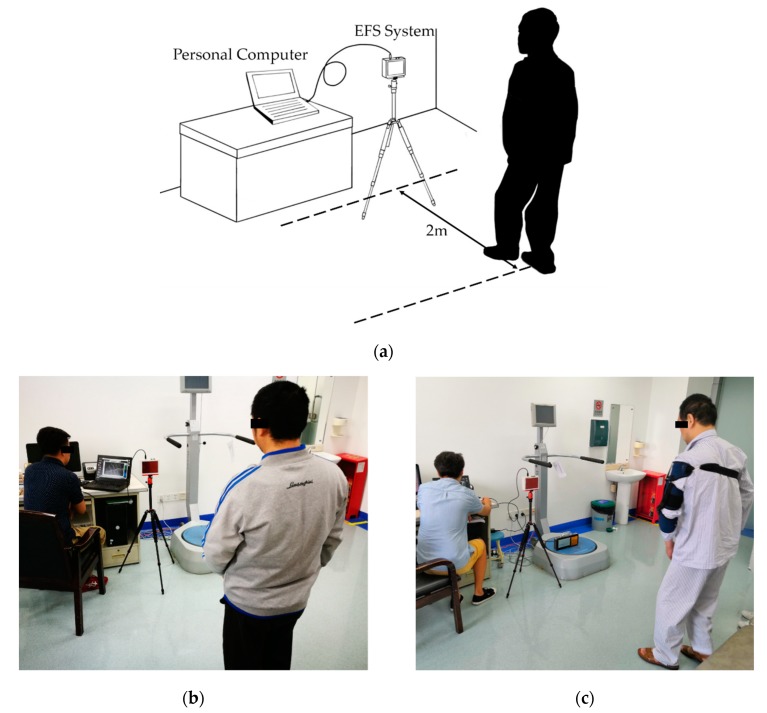
Illustration diagram and prototype of electrostatic measurement installation. (**a**) Illustration diagram of electrostatic measurement installation; (**b**) clinical test at the hospital; (**c**) clinical test at the hospital.

By adjusting the measurement installation sensitivity, the effective measurement distance of the installation was adjusted to 3 m. In order to reduce the measurement error caused by the angle between the human body and the electrode, the subject needed to maintain their coronal plane parallel to the electrode during the test. A diagram illustrating the electrostatic measurement installation is shown in Figure 1a. In order to reduce the influence of humidity on the printed circuit board, the circuit was placed in a waterproof sealed box. A clinical test at the hospital is shown in Figure 1b,c.

### 2.3. Experimental Conditions

In the experiment, subjects were required to walk continuously for at least 30 s at their most comfortable pace to collect enough gait data. In order to ensure the measurement effect, subjects were guaranteed a thorough rest during the measurement interval. Furthermore, subjects usually had an adjustment period before steady walking, so we removed 2 s of data at the beginning and at the end of each measurement to ensure data validity. At the same time, to eliminate the effect of temperature and humidity on the EFS method, the whole experiment was performed in the hospital laboratory with a temperature of 25 °C and relative humidity (RH) of 65%.

### 2.4. Feature Extraction

#### 2.4.1. Gait Symmetry 

Gait symmetry is a significant characteristic of human gaits, and neurological diseases such as stroke can significantly change one’s gait symmetry. To evaluate the parallels between the two lower extremities, gait symmetry can provide information about the control of lower limb movement that may be distinct from conventional features, such as temporal parameters, and may have a role in assisting the clinician’s treatment strategies. Unlike the customary gait symmetry equations, we used the dynamic time warping (DTW) distance to represent gait symmetry. DTW is a well-known method in the field of time series analysis, which is usually utilized to measure the similarity between two temporal sequences. The DTW algorithm calculates an optimal match between two time series and thereby computes their similarity through time shifting.

If one assumes that the gait electrostatic signal sequence generated by the subject’s left foot is ***L*** = {*l_1_*, *l_2_*,…, *l_n_*} of length *n*, the right foot sequence is ***R*** = {*r_1_*, *r_2_*,…, *r_m_*} of length *m*. The goal is to find an alignment between ***L*** and ***R*** with a minimal overall cost. Defining sequence ***W***= {*w_1_*, *w_2_*,…, *w_k_*}, where k is satisfied min(*n, m*) < *k* ≤ max(*n, m*). The *kth* element of ***W*** is defined as *w_k_* = (*i, j*)*_k_*, where *w_k_* is the Euclidean distance between *l_i_* and *r_j_*. DTW is the warping path with minimal total cost among all possible warping paths [24].

(1) $$DTW=\min\sum_{x=1}^{k}W_{x}$$

It can be concluded from the definition that if the gait signal sequence of the left foot is symmetrical with the right foot, the calculated result of the above formula will be a small value, and if they are asymmetrical, the value will be a large one. Therefore, the symmetry of the left and right foot is inversely related to the DTW value, which can therefore be utilized to represent gait symmetry.

#### 2.4.2. Complexity Characteristics

In information theory, entropy is a method of quantifying the degree of complexity in a signal. In this study, we used sample entropy (SampEn) to quantify the complexity of the gait signal [25,26], which was derived specifically for a biomedical time series, such as heart rate variability or an electroencephalogram [27]. The SampEn of a time series {*x(n)*} = *x(1)*, *x(2)*, …, *x(n)* of length *N* could be obtained through the following steps.

Step 1: M-dimensional vectors are formed as

(2) $$\{X_{m}(i)\}=x(i),x(i+1),\ldots ,x(i+m-1),1\leq i\leq N-m+1$$

Step 2: The distance between vectors ***X****_m_*(*i*) and ***X****_m_*(*j*) is defined as

(3) $$d[X_{m}(i),X_{m}(j)]=\max\left|x(i+k)-x(j+k)\right|$$

Step 3: Given a threshold *r*, $B_{i}^{m}(r)$ is defined as

(4) $$\left\{ \begin{array}{l} B_{i}^{m}(r)=\left\{No.of\;X_{m}(j)s|d_{chebyshev}(X_{m}(i),X_{m}(j)\leq r)\right\}\\ i\neq j \end{array}\right.$$

Step 4: Averaging over the series gives

(5) $$B^{m}(r)=\frac{1}{N-m}\sum_{i=1}^{N-m}B_{i}^{m}(r)$$

Step 5: For a limited series, the SampEn is defined as

(6) $$SampEn=-\ln\frac{B_{}^{m+1}(r)}{B_{}^{m}(r)}$$

In this study, we set *m* = 2, *r* = 0.2∗*std* as recommended by previous research [28], where *std* was the standard deviation of the time series.

#### 2.4.3. Stability Index

Gait sequence is a non-stationary, non-linear time series. Therefore, the conventional temporal gait parameters extracted from the time domain could not fully reflect its characteristics. The empirical mode decomposition (EMD) technique is a novel analytical method, which has been proven to be suitable for the analysis of nonlinear and non-stationary signals. The EMD method is a data-driven, adaptive, multi-scale, and robust signal processing method. Hence, it is suitable for processing nonstationary, nonlinear time series. In this study, we used the EMD method to analyze the frequency domain characteristics of gait signals, and a gait stability index calculation method was proposed. 

Each gait electrostatic signal was decomposed into a series of intrinsic mode functions (IMFs) using the EMD algorithm. Each gait signal sequence was divided into eight IMFs. Based on analysis of the dynamic patterns of the EFS gait signal and the IMFs, we inferred that the 3rd IMF and 4th IMF, denoted as IMF3 and IMF4, embodied the dominant patterns of gait frequency, while IMF1 and IMF2 embodied the high frequency fluctuations of the gait signal. We speculated that a “healthy” gait should have had more “energy” in key components of the gait signal itself and less high frequency “energy”. Therefore, subjects who walked with a more stable gait pattern would have had relatively high energy in IMF3 and IMF4 and low energy in IMF1 and IMF2. We calculated the instantaneous amplitude (IA) to quantify the ‘‘energy’’ of each IMF. The gait stability index (SI) proposed in this study was defined as

(7) $$SI=\frac{IA\;of\;IMF3+IA\;of\;IMF4}{IA\;of\;IMF1+IA\;of\;IMF2}$$

### 2.5. Classification Model

In this study, four machine learning techniques, i.e., the support vector machine (SVM), decision tree (DT), multilayer perceptron neural-networks (MLP) and k-nearest-neighbor (kNN), were applied because all of these techniques are often utilized to solve classification problems in nonlinear feature spaces and have been proven to be suitable for small size data sets, which was the same situation as this study.

SVM is a maximum margin-based classifier that constructs boundaries that maximizes the margins between the closest points and the hyper-plane dividing the two classes [29]. To create a linear separating hyper-plane for nonlinear problems, SVM employed kernel methods to map data to a higher dimensional feature space. Basically, SVM finds an optimal hyper-plane that separates the two classes of data sets based on the quadratic programming technique. In this study, the popular radial basis function (RBF) kernel was adopted and evaluated.

Decision trees are predictive decision support classifiers that create mapping from an input set to a target set [30]. Input features and machine-learning-based algorithms such as the ID3, C4.5 and C5.0 are used to construct a tree. This has the advantage of a simple structure, model interpretation and fast operation speed.

MLP is a widely used feed-forward artificial neural network model [31]. It is a powerful nonlinear classifier for learning complex nonlinear mapping from input to output variables. The MLP network consists of neurons that are arranged in three fully connected layers: (1) The input layer, (2) the hidden layer, and (3) the output layer. The MLP calculates the output values from a series of feature inputs, and the information moves in the direction from the input layers to the output layers through the hidden layers. In this study, MLP was trained by the back propagation algorithm, which adjusted the parameters of MLP by minimizing the least-mean-squares error between the output and the target.

kNN is a method of classifying a test sample by the labels of the k-nearest-neighbors of the test sample in the feature space [17]. kNN determines the label of the test sample by choosing the class that is most common among its k closest training samples. kNN is a type of lazy learning, which does not require estimating parameters and training before use.

In addition, to avoid overfitting and to make full use of the experimental data, the tenfold cross-validation method was applied for evaluating the generalization ability of the classifier. In the experiment, the area under the curve (AUC) was used to assess the performance of the classifier. A greater performance of the classifier always had a larger AUC value.

### 2.6. Analysis

As mentioned in Section 2.3, each test trial could acquire a gait electrostatic signal sequence with a length of 30 s, which was repeated five times for every subject, so 30 subjects received a total of 150 gait electrostatic signal sequences. Next, each gait sequence was normalized to eliminate the effect of amplitude on subsequent analyses. Three types of features were extracted from each gait sequence and used as an input to the classifier. Data analysis and the classifier were performed using Matlab (R2016b, MathWorks Inc., Natick, MA, USA). The mean and standard deviation (SD) of the features were compared between the hemiparetic patients (HP) and the healthy control (HC) group. By using SPSS Version 20.0 (IBM Corporation, Armonk, NY, USA), a Mann–Whitney test was applied to evaluate whether there was a significant difference between the HP and HC group in terms of each feature. The differences between the two groups were considered to be statistically significant if *p* < 0.05.

## 3. Results

**Figure 2:**
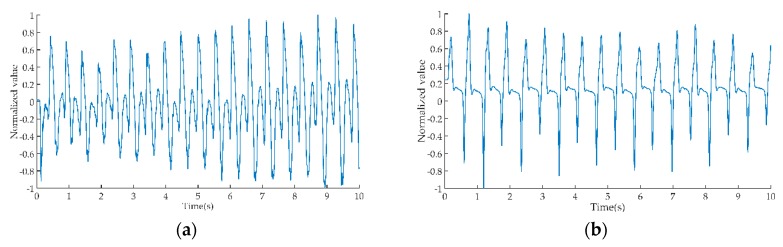
Time-domain waveform of hemiparetic patients (HP) and healthy controls (HC). (**a**) Gait electrostatic signal of HP; (**b**) gait electrostatic signal of HC.

In one test trial, the time-domain waveform of hemiparetic patients (HP) and healthy controls (HC) obtained by the EFS method is shown in Figure 2.

**Figure 3:**
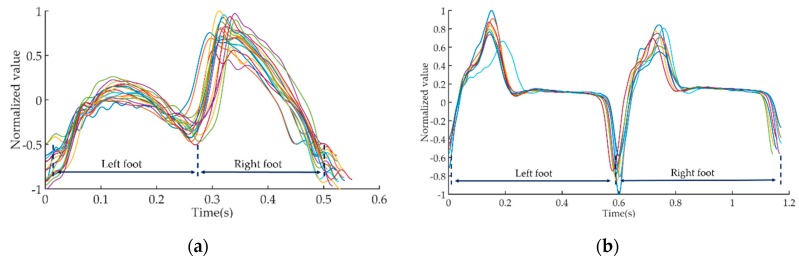
The illustration of gait cycle sequences. (**a**) Gait cycle sequences of HP; (**b**) gait cycle sequences of HC.

In order to facilitate the observation of signal characteristics, we extracted a 10 s gait signal from the full 30 s signal for illustration. Subsequently, we used the method proposed in the literature [20] to divide the 10 s time-domain waveform into several gait cycle waveforms. The local maxima point of the waveform coincided with the moments when the foot separated from the ground, the local minima point of the waveform coincided with the moments when the foot contacted the ground. Figure 3 shows a plot of all gait cycle waveforms of HP and HC in the same illustration.

The gait cycle sequences could be further divided into left foot gait sequences and right foot gait sequences, which were used to calculate gait symmetry by the DTW algorithm. 

**Figure 4:**
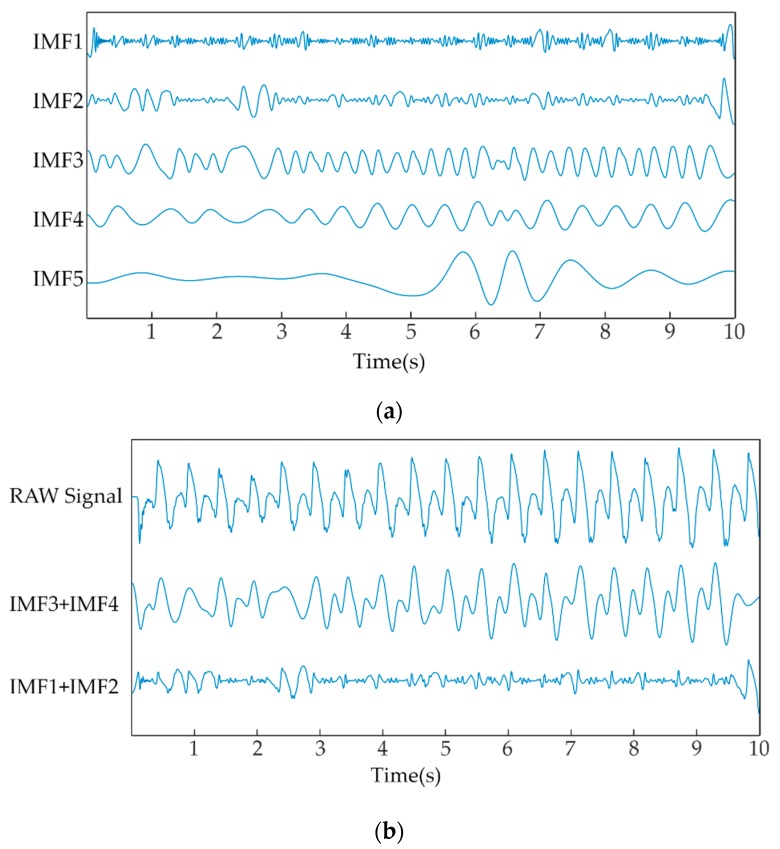
The intrinsic mode functions derived from a HP subject using the EMD method. (**a**) IMF1–IMF5 of the HP subject; (**b**) the IMF3 and IMF4 embodied dominant patterns of gait frequency and IMF3 and IMF4 embodied high frequency fluctuations of gait sequence.

Figure 4a shows the first five IMFs of the gait sequence of the same HP subject in Figure 2a. It was obvious that the five IMFs were exhibited from the relatively high frequency to low frequency components continuously. Figure 4b shows a raw gait electrostatic sequence of the same HP subject, and the dominant patterns of gait frequency and high frequency fluctuations of gait sequence obtained using EMD.

Table 1 shows the means and the standard deviations of the values of the three features for the gait electrostatic sequence for HP and HC subjects. From Table 1, it is obvious that for the feature of gait symmetry, the HC subjects had smaller means and standard deviations than HP subjects, which implied that HC subjects had a better gait symmetry and were able to maintain gait symmetry for a long period. In addition, from Table 1, it is also apparent that, compared with the HC subjects, the HP subjects had larger values of SampEn, which indicated that the gait electrostatic sequence of the patients were more variable. As for the stability index, Table 1 shows that the gait sequence of HP subjects contained more higher frequency energy than the HC subjects. The results were consistent with our assumptions that higher stability index (SI) values will reflect a more stable gait.

In our study, the Mann–Whitney test was implemented to test for statistical differences among HP and HC subjects and the results are shown in Table 1. The *p* values of the three features were all less than 0.05.

Table 2 shows the values of AUC for the classifications of HP and HC.

## 4. Discussion

Gait is a coordinated, symmetrical and rhythmic periodic motion, which can be disrupted by stroke. Hence, in our study, we used gait symmetry as a feature to describe patients with hemiplegia. We proposed a new approach to investigate gait symmetry by calculating the DTW distance between the gait electrostatic sequences of different lower extremities. The results in Table 1 show that the DTW distance of patients with hemiplegia is much larger than that of healthy control subjects. This is because the post-stroke patients could not control one side of their lower limbs and were unable to maintain normal height and speed during stepping, resulting in a difference in the gait electrostatic signal. Moreover, the standard deviation of DTW distance in the post-stroke patients was much larger than the healthy control subjects. This phenomenon can be expounded as a large change in the gait symmetry of the patient during a long-period of walking, and an inability to maintain the stability of the biped movement compared to healthy controls.

We believe that the symmetry of gait should maintain increased attention from both clinicians and researchers, because it may potentially have some negative associations. Asymmetrical gait can lead to difficulties in balance control, increased energy consumption, increased risk of falling and lower limb injury, and decreased activity capability. Therefore, it is crucial to find a method to acquire and analyze the symmetry of gait. The proposed gait electrostatic sequence-based DTW distance can be regarded as one of the alternative methods of quantifying the gait symmetry of post-stroke patients.

In order to quantify the difference between gait signal regularity, we utilized the SampEn algorithm to calculate the gait electrostatic sequence. Sample entropy, where larger entropy values indicate less regularity in a time sequence, has been widely used in pathological signal analysis [28]. Table 1 shows that the SampEn of HP subjects was significantly higher than the healthy control group, which indicated that gait signal periodicity of HP subjects during walking was worse than that of the healthy control group. In other words, the gait sequence of the healthy subjects had a higher degree of uniformity and monotonicity compared with post-stroke patients. In addition, the standard deviation of SampEn shows that, compared with healthy controls, the gait signal of post-stroke patients showed a more heterogeneous distribution. The results revealed that the inhomogeneous components of the gait sequences of the post-stroke patients were much stronger than the healthy control subjects. This might be due to the fact that the neuromuscular system is modified by the post-stroke syndrome, leading to gait variability change.

Previous studies have shown that fluctuations in healthy control subjects are relatively small and the variations of gait parameters are maintained at a lower level. However, for the post-stroke patients, the dynamics of gait are significantly changed, therefore, acquiring and analyzing the regularity change between post-stroke gait and healthy control gait is very meaningful.

As for gait stability, we developed an empirical mode decomposition-based stability index (SI). Our results suggested that this new index will be a potentially useful criterion for walking stability. Existing methods that reflect smoothness in gait sequence are generally based on spectral analyses, such as short-time Fourier transform (STFT) [19] or the autocorrelation function [32,33]. However, gait sequence is a non-linear and non-stationary time series, and the validity assumptions underlying these methods are debatable. In contrast, the EMD, and the proposed SI, is based on a self-adaptive method, where the data itself determine the boundaries and the signal is decomposed from high frequency to low frequency automatically without any a priori assumptions. Furthermore, in addition to the SI, the ability to investigate the individual intrinsic mode functions (IMFs) may enable researchers to explore specific variety in certain intrinsic scales of the gait patterns and their correlation with different pathology factors.

The results in Table 1 show that the relationships between the gait sequence and the intrinsic mode functions support the definition of the SI. The SI was significantly lower in post-stroke patients compared to healthy control subjects. This phenomenon can be explained as the hypothesis that a “healthy” gait pattern should have more “energy” within the key component of the step frequency (IMF3 and IMF4), and less ‘‘energy’’ within the high frequency components (IMF1 and IMF2). 

As shown in Table 2, the AUC value for the differentiation between post-stroke patients and healthy controls reached up to 0.94, obtained by the kNN classifier, which indicated that the proposed feature had a very strong discrimination ability. Compared with the literature [34], in which inertial sensors were utilized to acquire and analyze gait signals, our features and classifier reached a higher classification accuracy. Therefore, the proposed three types of features can effectively reflect the gait characteristics of post-stroke patients and has considerable potential for diagnosing post-stroke gait signals.

The present study still has some limitations. (1) Only 30 s of gait sequence data were used. The effect of sequence length on features should be considered in future studies. (2) The three proposed types of features were accurate in distinguishing patients from healthy controls, but whether the difference in the degree of disease could also be distinguished by these features remains to be further studied. (3) While the utility of these new features was supported by the results obtained during the experiment, a prospective study in a larger sample will be needed to assess the ability of these features.

Despite this limitation, the proposed EFS sensing-based gait signal analyzing method is still a promising method for assessing patient gait conditions. Due to the fact that post-stroke patients still need to complete exercise and rehabilitation at home after being discharged from hospital, our method could assist specialists in monitoring a patients’ rehabilitation status at home and correcting the rehabilitation exercise plan after evaluation.

## 5. Conclusions

In summary, we have presented a novel gait sequence analyzing method that uses the EFS sensing signal, which was based on three types of extracted features. The DTW algorithm, sample entropy method and empirical mode decomposition were utilized to obtain the symmetry, complexity character and stepping stability of the gait signals, respectively. The analysis showed that these features could potentially facilitate diagnosis of the hemiparetic gait. In addition, the experimental results of the classifier showed that these novel features had high predictive sensitivity. Therefore, our research suggests that gait symmetry, complexity characteristics and stepping stability should be given serious consideration in the study of a hemiparetic gait.

## Figures and Tables

**Table 1:** Means, standard deviations, and *p* values of the Mann–Whitney test of the three gait features for HC and HP.

	HP	HC	*p*-Value
**Gait Symmetry**	7.89 ± 4.17	3.45 ± 1.88	0.000
**SampEn**	2.56 ± 0.25	2.06 ± 0.09	0.012
**Stability Index**	0.59 ± 0.49	1.75 ± 0.56	0.000

**Table 2:** Value of AUC for the classifications of HP and HC.

	HP versus HC
**SVM**	0.91
**DT**	0.87
**KNN**	0.94
**MLP**	0.86
